# Intraoperative radiation therapy with 50 kV x‐rays: A multi‐institutional review

**DOI:** 10.1002/acm2.14272

**Published:** 2024-01-26

**Authors:** Anil Sethi, Sebastien Gros, Patrik Brodin, Beth Ghavidel, Xuedong Chai, Marija Popovic, Wolfgang Axel Tomé, Samuel Trichter, Xiaofeng Yang, Hualin Zhang, Valery Uhl

**Affiliations:** ^1^ Department of Radiation Oncology Stritch School of Medicine Loyola University Chicago Maywood Illinois USA; ^2^ Department of Radiation Oncology Montefiore Medical Center Albert Einstein College of Medicine Bronx New York USA; ^3^ Department of Radiation Oncology Winship Cancer Institute Emory University Atlanta Georgia USA; ^4^ Department of Radiation Oncology Stanford University School of Medicine Stanford California USA; ^5^ Department of Medical Physics McGill University Health Center Montreal Quebec Canada; ^6^ Department of Radiation Oncology Weill Cornell Medical Center New York‐Presbyterian Hospital New York New York USA; ^7^ Department of Radiation Oncology University of Southern California Los Angeles California USA; ^8^ Department of Radiation Oncology Summit Medical Center Emeryville California USA

**Keywords:** clinical implementation, IORT, kilo‐voltage therapy

## Abstract

This report covers clinical implementation of a low kV intraoperative radiation therapy (IORT) program with the INTRABEAM® System (Carl Zeiss Meditec AG, Jena, Germany). Based on collective user experience from eight institutions, we discuss best methods of INTRABEAM quality assurance (QA) tests, commissioning measurements, clinical workflow, treatment planning, and potential avenues for research. The guide provides pertinent background information and clinical justification for IORT. It describes the INTRABEAM system and commissioning measurements along with a TG100 risk management analysis to ensure safety and accuracy of the IORT program. Following safety checks, dosimetry measurements are performed for verification of field flatness and symmetry, x‐ray output, and depth dose. Also discussed are dose linearity checks, beam isotropy, ion chamber measurements, calibration protocols, and in‐vivo dosimetry with optically stimulated luminescence dosimeters OSLDs, and radiochromic film. Emphasis is placed on the importance of routine QA procedures (daily, monthly, and annual) performed at regular intervals for a successful IORT program. For safe and accurate dose delivery, tests of important components of IORT clinical workflow are emphasized, such as, dose prescription, pre‐treatment QA, treatment setup, safety checks, radiation surveys, and independent checks of delivered dose. Challenges associated with in‐vivo dose measurements are discussed, along with special treatment procedures and shielding requirements. The importance of treatment planning in IORT is reviewed with reference to a Monte Carlo‐based commercial treatment planning system highlighting its main features and limitations. The report concludes with suggested topics for research including CT‐based image‐guided treatment planning and improved prescription dose accuracy. We hope that this multi‐institutional report will serve as a guidance document on the clinical implementation and use of INTRABEAM IORT.

## 1 INTRODUCTION

Intraoperative radiation therapy (IORT) is a form of targeted radiation given to the tumor bed following surgical resection of the tumor.^1^, ^2^, ^3^ IORT is capable of precise dose delivery to tissues immediately surrounding the tumor cavity. IORT may be used anywhere in the body, for example, either as a sole radiation treatment for early‐stage cancer of the breast, skin, spine, brain, or when treating recurrent cancer in a previously irradiated site. IORT can also be used in combination with external beam radiation therapy (EBRT). For example, IORT may be given as a boost treatment at the time of tumor resection for many body sites including head and neck,^4^, ^5^, ^6^ brain,^7^ breast,^8^ abdomen,^9^ spine,^10^ pelvis,^11^, ^12^ and sarcomas.^13^, ^14^ Multiple trials have demonstrated safety and efficacy of IORT in controlling microscopic disease in the immediate vicinity of the treatment applicator. Due to high tumoricidal dose, reduced dose to normal tissues and patient convenience, the use of IORT has grown over the past two decades, especially for early‐stage breast cancer. The TARGIT‐A trial results were first published in 2010 with updates in 2013 and most recently in August 2020.^8^, ^15^, ^16^

Radio‐biologically, there are several distinct advantages of IORT. For example, IORT with low‐kV photon energy has a 1.5‐fold relative biological effect (RBE) compared to megavoltage electron beam due to its higher linear energy transfer (LET).^17^ IORT delivered by INTRABEAM (Carl Zeiss AG, Germany) uses 50‐kV x‐rays characterized by steep dose gradient that provides protection of nearby normal tissues. Treatment times are 20−55 min to deliver the prescribed single fraction dose of 20 Gy to the spherical applicator surface (or inner surface of the lumpectomy cavity) for breast cancer. During this long treatment time, cancer cells have a greater chance of DNA damage compared to the shorter treatment duration of other IORT units as well as EBRT.^18^, ^19^, ^20^ This longer treatment time also allows for the repair of single and double‐strand DNA breaks in normal cells.

Morrison et al. reviewed the USA National Cancer Database to analyze trends in IORT utilization for breast cancer.^21^ From 2004 to 2009, IORT accounted for 1.0% of all treatments, but from 2010 to 2014, its proportion increased to 4.0%, with a peak of 8.6% in 2014.

In order to safely and accurately deliver the large radiation dose within the time constraint of surgical procedure, IORT requires the combined expertise of a multidisciplinary team including a surgeon, a radiation oncologist, and a qualified medical physicist (QMP). This report, based on the collective experience of users from eight different institutions in North America, describes the technical aspects of the INTRABEAM IORT system and processes relevant to QMPs when establishing a safe and effective IORT program in a radiation oncology department. It also reviews special circumstances where the physical and dosimetric aspects of the INTRABEAM IORT system differ from external beam radiotherapy protocols for particular clinical situations and how to address them.

Although this report uses breast IORT with spherical applicators as an example, readers are advised to adapt their clinical workflow and checklists for other anatomic sites and appropriate treatment applicators as needed.

## 2 DESCRIPTION OF INTRABEAM SYSTEM: UNIQUE FEATURES AND DOSIMETRIC ADVANTAGES

**FIGURE 1:**
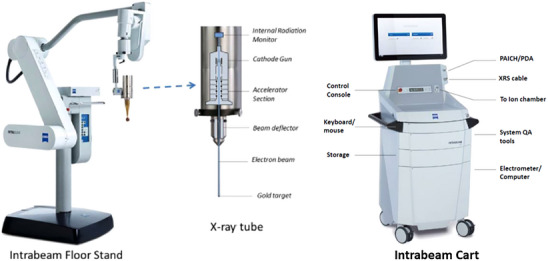
(a) Left: INTRABEAM NC32 floor stand and x‐ray source shown with a spherical applicator attachment. The robotic stand provides submillimeter precision and accuracy of movement for the x‐ray tube in six dimensions (translation and rotation. (b) Right: INTRABEAM 600 treatment unit showing major components: treatment console, computer for dose recording and verification, ion chamber, electrometer, and accessories and tools for quality assurance (QA) measurements. Figure adapted from Reference 33.

The IORT process reviewed in this report is based on the INTRABEAM PRS 600 (photon radiosurgery system PRS, INTRABEAM, Carl Zeiss Surgical, Oberkochen, Germany) which includes a low‐energy x‐ray source (XRS) emitting 50 kV photons at a high dose‐rate (Figure 1).^8^, ^22^, ^23^, ^24^, ^25^, ^26^, ^27^, ^28^, ^29^ An overview of IORT systems including their clinical applications and safety considerations was provided by the ASTRO emerging technology committee report on electronic brachytherapy.^30^ A description of the INTRABEAM system is also available from the product documentation provided by Zeiss.^31^, ^32^, ^33^

The source of therapeutic photons used in INTRABEAM is a narrow beam of electrons accelerated in an x‐ray tube before traveling through a 10 cm‐long drift tube with a diameter of 3.2 mm. The electrons strike a very thin (1 μm) hemispherical gold target located at the distal end of the drift tube to generate low‐energy x‐rays (50kVp, 40 μA) in isotropic directions. This design results in some of the x‐rays scattering back upstream into the drift tube towards an internal rate meter (IRM) that continuously monitors the dose rate for constancy during treatment (Figure 1a). The IRM provides an indirect measure of the dose rate in real‐time delivered at the probe tip. For patient treatments, a treatment‐specific applicator is attached to the x‐ray tube followed by pre‐treatment QA and calibration check of the device. These QA tests, described in more detail in later sections, verify mechanical alignment of the probe, electron beam steering and x‐ray radiation isotropy, dose linearity, dose rate constancy, and output check. Treatments may be paused and restarted as needed.

For treatment planning and personnel radiation protection, due considerations must be given to the beam quality of the XRS. Although the nominal energy of radiation from the INTRABEAM device is 50 kV, the energy spectrum (bare probe) includes a significant component of the lower energy x‐rays (∼10–20 kV).^25^ These low energy x‐rays are easily absorbed in the surrounding medium resulting in a hardened beam. The exact effective energy of resultant x‐rays depends on the specific treatment application, the applicator used, the treatment depth, and tissue heterogeneity. Typical HVLs range from 0.1 mm of Al (unfiltered beam in air or bare probe without an applicator) to 1−2 mm Al (spherical applicator) at 1 cm depth in tissue. The mean energy of bare probe x‐rays is estimated to be ∼21 kV and those from spherical applicators between 29 and 30.85 kV.^34^ For radiation protection and shielding determination, an effective energy of 30 kV is considered appropriate.^35^

The advantages of INTRABEAM IORT are its ability to deliver a large radiation dose (10–20 Gy) to a target volume with rapid dose fall‐off and hence limited exposure to adjacent organs at risk (OARs). Another advantage is the direct visualization of the tumor bed and the ability to move nearby critical structures away from the target area to further minimize dose delivered to OARs. Additionally, with appropriate precautions, such as, the use of portable personnel shielding, lead aprons and flexi‐shields around the treatment site, the low‐energy x‐rays allow IORT delivery in standard hospital surgical suites with minimal radiation exposure risk to the personnel.

Recent IORT advances, such as, the availability of a variety of treatment applicators to shape radiation dose to the desired target volume have resulted in significant gains in IORT clinical applications.^10^, ^36^, ^37^, ^38^ The INTRABEAM system can be commissioned and used with spherical, flat, surface, or needle applicators.^29^ Owing to their symmetrical shape, spherical applicators are used to deliver a uniform dose to the breast lumpectomy cavity or for intra‐cranial treatment applications.^39^ Spherical applicators are made of polyetherimide or thermoplastic (C37H24O6N2) material (ULTEM, transition temperature = 216°C) that is capable of resisting high temperatures during sterilization procedures (typically ≤138°C). Figure 1a shows the IORT system with a spherical applicator attached to the x‐ray tube mounted on a robotic stand. The latter is equipped with weight compensation and electro‐magnetic brakes to ensure flexible and precise applicator positioning with sub‐millimeter accuracy. The surface and flat applicators are encased in stainless steel with thermoplastic end for radiation delivery, making them ideal when a uniform planar dose is desired at a given tissue‐depth. Surface and flat applicators are designed to yield a uniform dose at target surface and 5‐mm depth in tissue respectively. The needle applicator is specially designed for kypho‐IORT in spine metastases.^10^, ^36^ The needle applicator creates a spherical dose distribution at the probe tip for irradiation of surrounding tumor volume. All INTRABEAM applicators are available in a range of sizes to enable custom IORT treatments depending on the location and extent of target area (Table 1).

**TABLE 1:** Types of treatment applicators available with INTRABEAM IORT and their usage.

Applicator type	Spherical	Flat	Surface	Needle
Applicator size (diameter, mm)	15, 20, 25, 30, 35, 40, 45, 50	10, 20, 30, 40, 50, 60	10, 20, 30, 40	4.4
Prescription dose specification	Applicator surface	*d* = 5 mm	*d* = 0 mm (surface)	*d* = 5 mm
Anatomical sites	Breast, brain, intra‐cavitary applications	Head and Neck, abdomen, pelvis	Superficial, skin tumors	Spine metastases, brain, and interstitial applications

Although spherical applicators have been in use for more than two decades for breast IORT, flat, surface and needle applicators have been recently added and their clinical applications are evolving.^38^, ^40^ However, clinical data for these applicators are still scarce.

Treatment sessions are controlled by an integrated treatment delivery unit, a recent model of which is INTRABEAM 600 (Figure 1b). Major system components include a control console to record and verify treatment parameters and communicate with the XRS unit. In addition, the system houses a full complement of dosimetry and QA tools (described later in this section), a dedicated electrometer, ion chambers, and connection cables to the XRS. The compact and integrated system design of the INTRABEAM 600 affords an efficient and streamlined workflow. A custom‐designed water tank is available from Zeiss to assist the user in performing system QA and commissioning checks.^31^, ^32^ The shielded water tank allows high precision and accurate (<0.1 mm) movement of the x‐ray probe tip for dose measurements. The INTRABEAM system is also equipped with a 3D treatment planning system, Radiance treatment planning system (TPS). With patient computed tomography (CT) data, the Monte Carlo‐based treatment planning system is capable of computing tissue heterogeneity corrected 3D dose distributions for target and adjacent critical structures (see Section 6).

## 3 SYSTEM COMMISSIONING

Prior to initiating commissioning measurements, a radiation protection survey of the operating room (OR) designated for IORT is mandatory. These measurements are typically performed in a simulated treatment geometry using a water phantom. Exposure levels are measured with a radiation survey meter at various points inside and outside the treatment room. Particular attention needs to be given to all OR entrances that staff may occupy during long irradiation procedures. In general, medical physicists tasked with such radiation protection surveys may not be intimately familiar with the layout of the operating room suits. Architectural drawings of the OR layout may be used to identify all potential points of interest, including access doors and surrounding areas with occasional and regular occupancy. For instance, it may be important to identify unused observation galleries separated by glass windows that may be hidden from immediate view. Similarly, there may be rest areas and storage rooms with gurneys that could be used by the OR staff during breaks and rest periods, and this may result in inadvertent radiation exposure.

The radiation protection survey is followed by IORT commissioning measurements. The latter include comprehensive testing and quality assurance of all delivery equipment along with beam data acquisition and validation.^41^

The commissioning process may be divided into two broad categories: mechanical and radiation checks. Mechanical tests include the integrity checks of cables, QA devices, treatment applicators, robotic x‐ray stand (including stand balancing and precision positioning tests), and software related to QA tests. An important mechanical test of x‐ray probe straightness needs to be done prior to performing any dosimetric measurements. This mechanical test verifies that the probe is straight within <0.1 mm. If the probe were suspected to be bent, further dosimetric tests would not be successful.

As with EBRT machines, beam data measurements and validation are two of the most important components of IORT commissioning since they ensure the accuracy of patient‐delivered dose. Although not mandatory, these tests are strongly recommended as they not only allow the physicist to gain familiarity with the IORT system, but also understand its strengths and limitations. Of note, the IORT beam data commissioning work includes both in‐air and in‐water measurements. Additional dose measurements may be needed with various treatment applicators attached to the x‐ray tube. These require the use of appropriate radiation detectors (ion chamber, film and OSLDs) and phantoms (water tank and solid water slabs).

Radiation tests consist of x‐ray dose output and percent depth dose measured in water with a thin window plane parallel plate ion chamber that is appropriate for use with low energy x‐rays. These measurements are performed in a radiation‐shielded water tank with two orthogonally placed ion chambers in waterproof plastic ion chamber holders.^31^, ^32^ A motor with micrometer controls allows precise XRS positioning (±0.1 mm) relative to ion chambers. In addition, the XRS may be rotated around the long axis of the probe to check for beam isotropy. These features of the water phantom permit the user to perform complete array of required commissioning measurements in water. In the absence of a water phantom, above tests may be conducted in a solid water slab phantom and radiochromic film.

Other radiation measurements include a check of the x‐ray beam positioning (dynamic offsets <0.1 mm from center), dose isotropy (<5%), beam flatness/symmetry (for flat and surface applicators), and dose linearity and reproducibility (<1%). All of these tests have been described in detail by Muralidhar.^41^

## 4 QUALITY ASSURANCE

Zeiss requires that daily or pre‐treatment QA tests be performed prior to conducting in‐water measurements. Daily QA procedures involve testing and validation of mechanical integrity and dosimetric accuracy of the system. These tests include verification of x‐ray probe straightness, dynamic offset, photo diode array (PDA) source check, and probe adjuster and ion chamber holder (PAICH) output check measurement.

The PDA consists of five diodes positioned orthogonally to each other to permit check of radiation isotropy of the emitted x‐rays. The PAICH device equipped with an ion‐chamber insert is used to verify XRS output.

Routine quality assurance is an integral component of a safe and accurate radiotherapy system. For the INTRABEAM unit, the requirements are more stringent due to two reasons: the unique mechanical design of the x‐ray probe and treatment applicators and the use of low energy x‐rays (50 kV) at very high dose rates (>5Gy/min). At the time of publication by Eaton in 2012,^28^ there were very few reports available in the literature documenting independent verification requirements of the INTRABEAM system. Whereas detailed QA steps are given below, a summary of the required tests are listed by Eaton.^28^ A more recent publication, AAPM TG‐182,^42^ addressed risk assessment specific to IORT based on the methodology proposed by AAPM TG‐100^43^ (see Section 7). AAPM TG‐182 report includes detailed process maps and a *Failure Modes and Effects Analysis* (FMEA) for two commercially available IORT systems: Xoft and INTRABEAM.^42^

In the United States, the Conference of Radiation Control Program Directors (CRCPD) published a guidance document for individual states to regulate the use of electronic brachytherapy (EB) devices.^44^ At the time of this publication, INTRABEAM was one of only two EB devices available in the United States. Most states have now released regulatory requirements for the use of EB devices. Although the regulations may vary by state, expectations regarding the radiation safety and quality assurance programs follow CRCPD guidance. Quality assurance (QA) tests must be performed pre‐treatment (i.e., on the day of treatment), annually, and at regular intervals not exceeding 6 months. The training of authorized users (AUs) and qualified medical physicists (QMPs) should recur annually following the initial training provided by the manufacturer. Example state regulations are given in refs.^45^, ^46^

### 4.1 Pre‐treatment/daily QA

Pre‐treatment QA and verification tests occur prior to all IORT patient treatments with INTRABEAM. Although these QA tests may be completed any time within a 36‐h window prior to treatment delivery, it is recommended that the tests be performed on the day of treatment. An example of the recommended steps for IORT pre‐treatment quality assurance is described in Appendix 1.

There are four tests included in the daily QA procedure. Two of them are *mandatory* (*M*) whereas the others are *recommended* (*R*): (i) PDA source isotropy check (*M*); (ii) PAICH output check (*M*); (iii) probe adjuster test (*R*); and (iv) dynamic offsets (*R*).

These daily QA tests should be performed in the proper sequence, as given in Appendix 2. If any of these QA checks fails to complete, all tests must be verified.

### 4.2 Monthly QA

While there is no specific regulation for the required frequency of periodic QA tests, performing monthly QA is important for the centers where the time interval between treatments may exceed 4−5 weeks. During monthly QA, the verification of the source parameters in the treatment computer is performed. For example, the measured dose rate after a PAICH test may be compared to a manual calculation of the dose rate based on the bare‐tip source dose rate table. The ratio of dose rates should match the values reported by the system after a PAICH test. The dosimetry of the system should also be tested periodically. As a constancy check, a simple treatment plan can be delivered in water, to measure the dose at some representative point. The dose measured in water with a calibrated ion chamber is next compared to the system‐estimated dose and to a manual calculation. The accuracy of the treatment delivery time may be verified with an independent timer or a stopwatch. Additional QC checks may include verifying the availability of sterile drapes for the INTRABEAM stand and breast shield supplies. An example monthly QA procedure is given in Appendix 3.

### 4.3 Annual QA

Annual QA typically follows XRS installation at the user site after factory maintenance and re‐calibration by Zeiss. The source installation is performed by a Zeiss service engineer who updates new source calibration data files as well as any ion chamber or electrometer coefficients into the control computer. Following the source installation, the service engineer completes four daily QA tests listed above in Section 4.1.

Subsequently, additional manufacturer‐mandated tests related to source change are performed at the user site. These include verification of the x‐ray beam output in water at 2 cm from the source, with a tolerance of 5.2%.^47^ The water tank provided by the vendor allows the measurement of depth doses (with a 0.1 mm precision) along the central axis as well as at 90° from the probe axis.^32^ It is recommended to perform a depth dose measurement over the full range of depths reported in the calibration file. The source isotropy may be verified with the lateral ion chamber holder. The source can be rotated and set to eight equally spaced azimuthal positions. Other detectors to measure source isotropy may include film and OSLDs.

A summary of annual QA tests is given below:

1. Test new source output in water phantom against factory measurements to be within 5.2% (per manufacturer).
2. Verify percent depth‐dose‐rate against manufacturer‐generated table (not mandatory but recommended).
3. Verify relative angular dose distribution from source—isotropy to be within 5%.
4. Perform simulated treatment in water phantom to verify delivery time and timer accuracy.
5. Verify electrometer calibration certificate is correctly entered in the control console.
6. Verify new ion‐chamber calibration certificate is correctly entered in the control console.
7. Verify source position accuracy from PAICH measurements.
8. Test accuracy of transfer functions (TF) required to convert in‐air measured dose to that delivered with the applicator. These measurements may be conducted annually with different applicators to sample entire user inventory. Run a simulated plan in water and calculate dose from the charge reading using calibration V4.0 protocol.

Material and inventory

1. Verify applicator inventory.
2. Verify condition of XRS, robotic stand, and all applicators.
3. Verify number of accumulated sterilization cycles for each applicator.

## 5 CLINICAL CONSIDERATIONS

### 5.1 Clinical workflow

A well‐understood and streamlined clinical workflow is essential for safe and accurate IORT delivery. For breast IORT, the workflow closely follows that of the surgical OR team, leading up to the lumpectomy tumor excision. Prior to the surgical procedure, the IORT team ensures that correct patient information and treatment consent has been obtained. Depending on the location and laterality of the tumor, the IORT stand may be placed on either side of the patient.

To prepare for the IORT case, the medical physicist performs the pre‐treatment QA. This consists of ensuring the XRS output (Gy/min) and anisotropy are within tolerance (see Section 4). Next, the XRS is mounted on the IORT stand and is connected to the cable from the stand. Verification of cable connections is important as failure to do so prior to sterile draping of the stand will lead to significant treatment delays as the stand and treatment unit will need to be re‐draped. Lastly, the physicist secures the cable connection between the floor stand and the IORT cart.

Following tumor excision, the surgeon examines the lumpectomy cavity together with the radiation oncologist to decide on the appropriate applicator size for the treatment. A sterile ruler may be used to measure the cavity diameter. An alternative strategy proposed by the team at Montefiore uses two sets of stainless steel dummy applicators designed to mimic the size of the IORT spherical applicators.^48^ The sterile stainless steel applicators are used by the OR team to determine which applicator would best fit the surgical cavity.

After the applicator size has been determined, the serial number of the selected spherical applicator is confirmed and the applicator mounted to the stand followed by the sterile draping procedure. The INTRABEAM robotic stand is then moved into position next to the patient and the applicator secured in the lumpectomy cavity by the surgeon and the radiation oncologist. The surgeon then raises tissue flaps around the applicator to create a skin bridge and the applicator‐to‐skin distance is measured using ultrasound at the superior, inferior, medial and lateral sides of the applicator. Flexible lead/tungsten shields are placed covering the applicator and breast to minimize any stray radiation to the OR personnel during IORT procedure.

Next, the medical physicist generates a treatment plan and calculates treatment time to deliver 20 Gy to the applicator surface or the inner surface of the breast lumpectomy cavity. An independent calculation is highly recommended as a QA check to confirm treatment time accuracy before commencing IORT. This can be done using a simple calculation formalism or with the help of look‐up tables based on the dose‐rate measured during pre‐treatment QA. Once treatment parameters are confirmed, the radiation oncologist initiates the treatment, and the medical physicist monitors the XRS output and isocentricity during treatment delivery.

**FIGURE 2:**
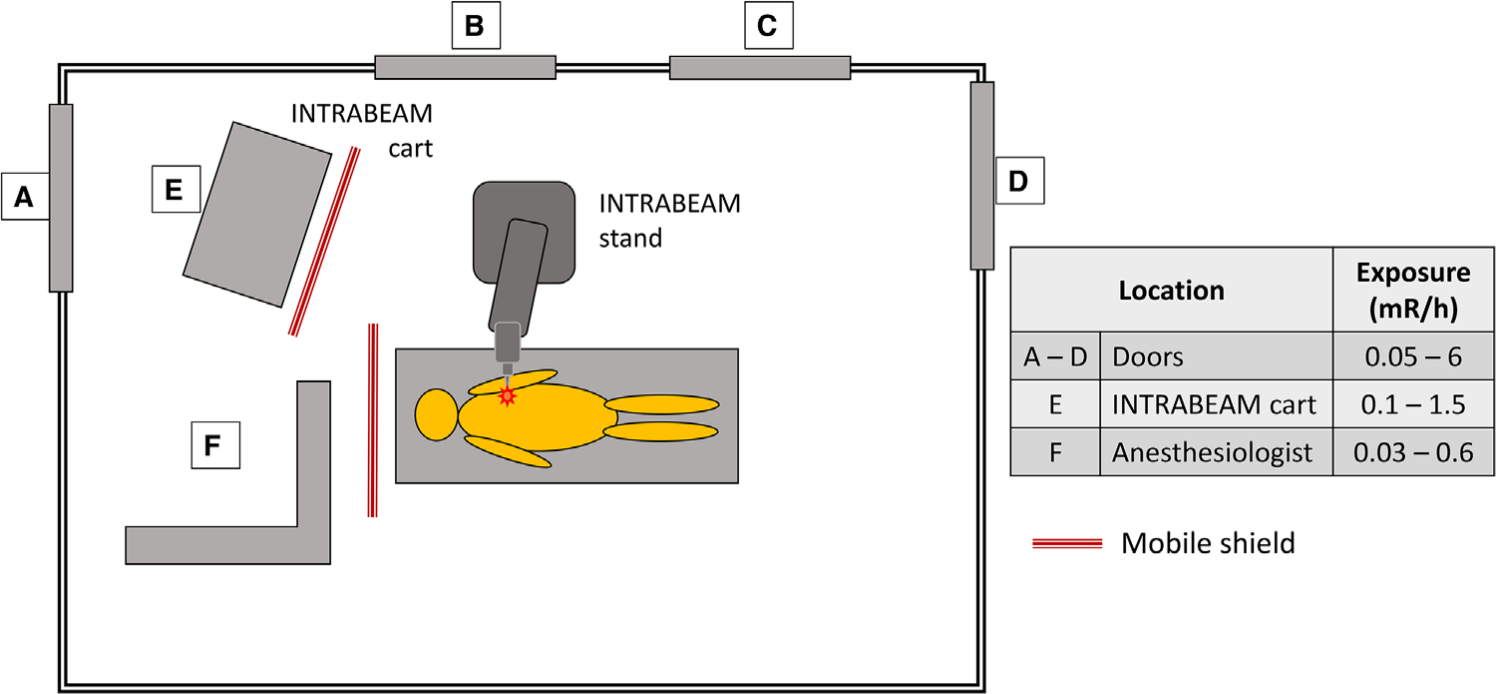
A layout of typical operating room (OR) and radiation exposures encountered during intraoperative radiation therapy (IORT) delivery. (1 mR/h = 0.01 mSv/h).

Depending on state regulations, a radiation survey of the OR may need to be performed in areas around the patient within the room as well as outside and dose rates documented. Based on our collective experience, unshielded exposure rates at 1 m from the bare probe have been measured to be up to 1 R/h. which are consistent with those reported by Eaton et al.^35^ It is important to ensure that appropriate mobile shielding, lead aprons, and radiation signage are in place to limit exposure to OR personnel. An OR layout with typical radiation exposures encountered during IORT delivery is shown in Figure 2. With appropriate shielding and precautions, all personnel exposure measured during treatment delivery is well within recommended guidelines. These readings are in the range that has been previously reported.^35^ Nonetheless, the operating room must be designated a controlled area with limited access.

Once treatment is completed, the surgeon frees the treatment applicator from the lumpectomy cavity. The medical physicist will then move the INTRABEAM stand away from the sterile field, remove the applicator and drape, and unmount the XRS from the stand. The stand, the XRS tube and accessories are cleaned with moist wipes and disinfected with fast‐acting alcohol surface disinfectant.^47^ Depending on the OR clinical schedule, the physicist can either safely store the IORT equipment or initiate a new pre‐treatment source QA for the next patient.

The applicator sterilization process needs to be developed and implemented keeping in mind IORT clinical schedule and available resources. The applicators must be cleaned, disinfected, and sterilized with steam and air‐dried following each procedure as per vendor instructions.^47^ The sterilization is done for a minimum of 5 min at temperatures of 132–135°C. Per manufacturer guidelines, all INTRABEAM applicators have finite number of clinical uses limited by the maximum number of sterilizations allowed: spherical applicators can undergo 100 sterilization cycles while both flat and surface applicators are restricted to a maximum of 50 sterilizations. Therefore, it is advised that the sterilization cycle history for each applicator be tracked independently by each user. For example, a spreadsheet listing each applicator and their unique serial number can be updated following the IORT procedure to reflect the number of allowed sterilization cycles remaining. Good practice recommends the physicist to verify remaining sterilization cycles as part of annual QA (Table 2).

**TABLE 2:** Summary of quality assurance (QA) recommendations for the INTRABEAM system.

Tests	Tolerance
**Daily QA**	
Probe adjuster test	0.1 mm^a^
Dynamic offset	0.1 mm^a^
PDA source check	Pass
PAICH output check	1% from baseline^b^
System dose rate versus table	1% from baseline^b^
**Monthly QA**	
**INTRABEAM system tests**	
Temperature probe (independent check)	2°
Pressure sensor (independent check)	1%
PDA source check	Pass
PAICH output check	1% from baseline^b^
Dosimetric accuracy	5%
Timer accuracy	5%
***Equipment and supplies***	
Radiation monitor calibration date and battery level	Per protocol/functional
Adequate supply of sterile drapes and breast shields	Functional
**Annual QA**	
***Source and System***	
Verification of calibration coefficients in system	Pass
XRS output	5.20%^c^
Depth dose rate	5% (10% for depth <10 mm)
Isotropy	5%
XRS dosimetric accuracy	5%
Timer accuracy	5%
Applicator dose measurement (one of each type, spherical, flat and surface. Rotate applicators annually)	5%
***Material and inventory***	
Applicator inventory	All applicators accounted for
Condition of source, stand and applicators	Good
INTRABEAM stand operation	Functional and balanced
Verify number of sterilization cycles	Spherical: <100
	Flat/surface: <50

Abbreviations: PAICH, probe adjuster and ion chamber holder; PDA, photo diode array; XRS, x‐ray source.

^a^ As required.

^b^ Baseline established after source exchange/commissioning.

^c^ Per manufacturer recommendation.

### 5.2 In‐vivo dose measurements

One of the major benefits of IORT compared to EBRT for breast cancer is the reduced dose to the surrounding tissues and better skin cosmesis, attributed to considerably lower skin dose.^49^, ^50^ Because of the standard prescription dose of 20 Gy to the applicator surface and a lack of computerized treatment planning, the dose to the skin and other organs‐at‐risk (OARs) may be difficult to estimate. Thus, in‐vivo dosimetry with radiochromic film^51^, ^52^, ^53^ and thermoluminescent dosimeters (TLDs)^54^, ^55^ provides two distinct advantages to an IORT program; (i) quality control in terms of estimating whether the delivered dose matches expectations, and (ii) a means of estimating OAR doses from the IORT procedure. The main challenges with in‐vivo dosimetry include the use of low energy (50 kVp) x‐rays that require specific calibration, the steep dose gradients from the applicator surface, and the need for measurements to be performed in a sterile OR environment. This section will focus on in‐vivo dosimetry performed using a variety of techniques, and the different methodologies that have been developed to tackle the challenges mentioned above.

An Italian group designed an in‐vivo dosimetry program based on EBT2 radiochromic film to measure the dose to the tumor bed, skin surface, and pectoral muscle for left‐sided cases.^52^ The film batches were calibrated specifically for IORT measurements with INTRABEAM, and pieces of film were wrapped in sterile pouches for in‐vivo measurements for a patient. Based on 23 individual measurements they found an average dose of 13.52 ± 1.21 Gy between the applicator and target breast tissue, as well as on average 2.22 ± 0.97 Gy at the skin surface 1–2 cm from the applicator surface.

An alternative approach to in‐vivo skin dosimetry methodology is based on optically stimulated luminescence dosimeters (OSLDs). The method involves calibrating OSLDs in air to the response of the INTRABEAM 50 kVp XRS using a 5 cm spherical applicator with OSLDs placed at each cardinal angle, using the v.4.0 protocol to deliver a known dose at the applicator surface.^56^ OSLDs were sterilized and placed on patients’ skin where the surgeon performed ultrasound skin bridge measurements. Using measured data from 25 IORT treatments, a model for estimating skin dose as a function of the applicator‐to‐skin distance was developed, and validated on subsequent five consecutive treatments. The average skin dose measured from the 25 IORT treatments was 1.18 ± 0.88 Gy at an average skin bridge distance of 19.9 ± 5.1 mm, with *D*_min_ = 0.17 Gy and *D*_max_ = 4.77 Gy.

Another method for skin dose measurements was suggested by a group in Malaysia, where they used Monte Carlo simulations to estimate the skin dose during INTRABEAM IORT, with calculations verified by EBT3 film measurements.^57^ The authors simulated skin dose for applicator‐to‐skin distances from 0.5 to 3.0 cm, with spherical applicators ranging from 1.5 to 5.0 cm diameter. They found that higher skin doses were estimated with larger spherical applicators (≥4.0 cm), and that a skin bridge distance greater than 1.0 cm may be necessary to keep the skin dose <6 Gy in those cases.

In general, these reports agree that applicator‐to‐skin distance of >1.0 cm will result in a skin dose low enough to be considered safe (<5–6 Gy). The presented methodologies for in‐vivo dosimetry could also be used for QA purposes to ensure that the expected dose was delivered at a given distance from the IORT applicator surface.

## 6 TREATMENT PLANNING

### 6.1 Current treatment dose calculation

Although both IORT and EBRT share common goals, the treatment planning for IORT has different technical requirements than EBRT. For example, the treatment region for IORT is typically undefined until after surgical resection is complete. Imaging is a prerequisite of EBRT planning, but imaging capabilities in the OR setting are limited, and current OR imaging solutions do not offer the same precision as the gold standard CT simulation.^58^ Current INTRABEAM treatment planning model utilizes a method for calculating delivered doses based on the 1‐dimensional fit of a look‐up table of depth dose rates measured in water along the central axis of the XRS. The effect of treatment applicators is accounted for by using a depth‐dependent correction factor or transfer function (TF) that converts the measured dose in water with a bare probe to that with the applicator in place. Whereas this method provides a quick dose calculation, it has several limitations, namely: (1) the water‐based measurements do not account for tissue heterogeneity; (2) for the nonspherical FLAT and SURFACE applicators, the 1‐dimensional depth dose measurements assume a uniform surface dose distribution and do not account for the divergence of the photon beam; (3) the current source calibration method employed by Zeiss for dose calculation needs validation.

### 6.2 Radiance TPS

**FIGURE 3:**
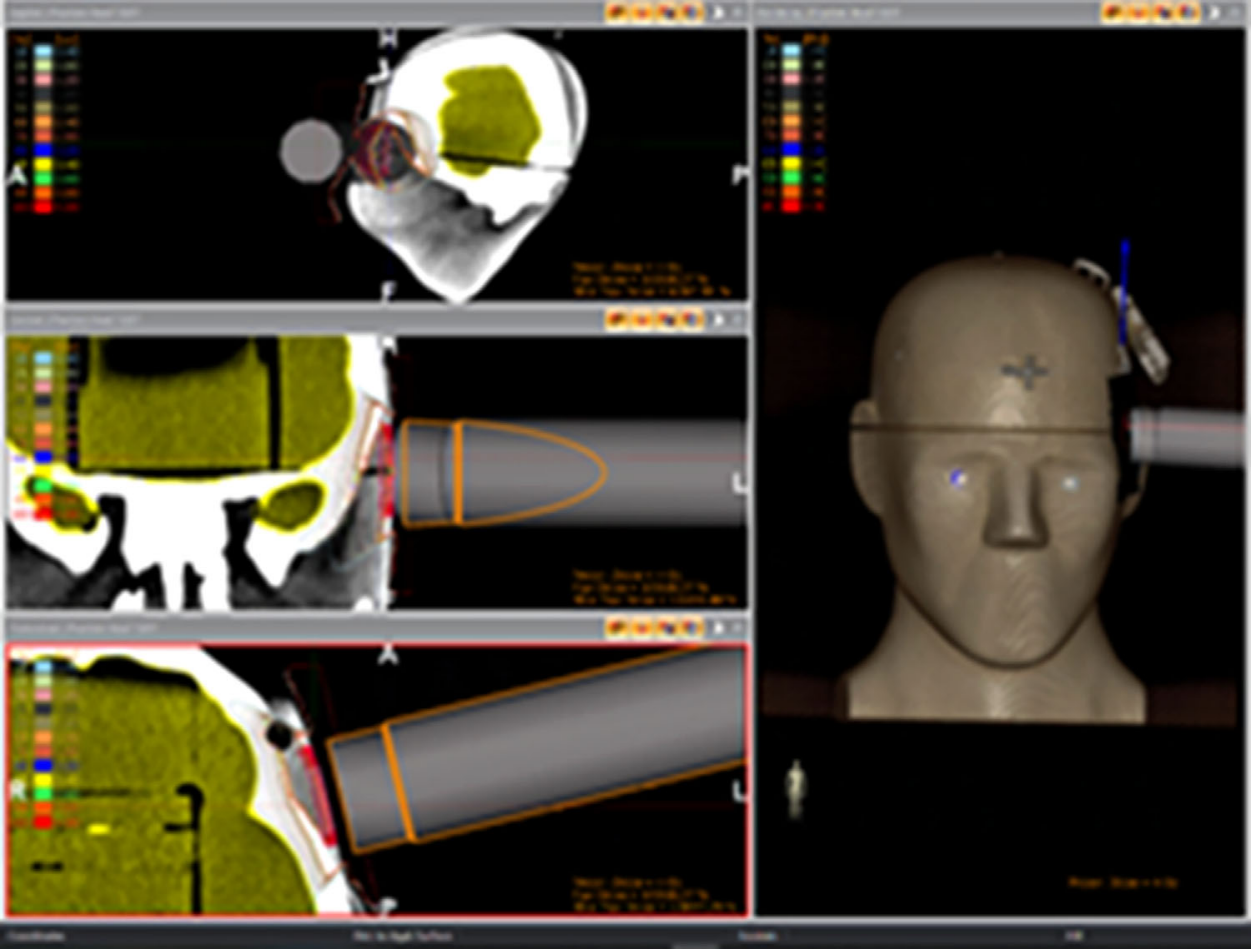
Radiance treatment planning system (TPS) user interface for treatment planning illustrating the positioning of a simulated flat applicator to plan a superficial scalp irradiation in an anthropomorphic head phantom.

The INTRABEAM 600 model is equipped with the *Radiance* TPS initially developed for electron‐based IORT (IOERT).^59^ As such, pre‐operative images can be loaded for treatment planning and dose calculation. The TPS provides contouring tools to segment volumes of interest in IORT such as target volumes (GTV/CTV/PTV) intended for irradiation and any surgically resected volumes and OARs. The Radiance TPS allows users to select and position a range of virtual applicators to fit specific radiation delivery geometry and to create resection cavities in order to simulate the surgical conditions of IORT irradiation. The dose engine implemented in Radiance TPS employs a hybrid Monte Carlo algorithm to model the INTRABEAM XRS and simulate photoelectric and Compton interactions in the low energy (<50 kV) x‐ray range. Dose calculations displaying 3D dose distribution and dose volume histograms (DVH) may be performed in either homogeneous (water) or heterogeneous (in medium) conditions using the density information from CT images. Figure 3 shows an example of the Radiance TPS interface.

### 6.3 TPS Commissioning/QA

While there are no published recommendations specific to the commissioning of TPS for low energy XRSs, the methodology outlined in AAPM TG‐72 report^60^ for the commissioning of the beam characteristics of the radiation source may be used. Additionally, guidance to test specific capabilities of the TPS can be found in AAPM MPPG 5.a.^61^ Validation measurements of XRS dose rate tables provided by Zeiss in water were highlighted in Section 4.

#### 6.3.1 Needle and spherical applicators

The measurements for the needle and spherical applicators can follow the same methodology developed for the bare source to compare measurements in water against transfer function tables provided by Zeiss. Isotropy of source emission can be verified in water with films or ion chambers.

#### 6.3.2 Flat and surface applicators

**FIGURE 4:**
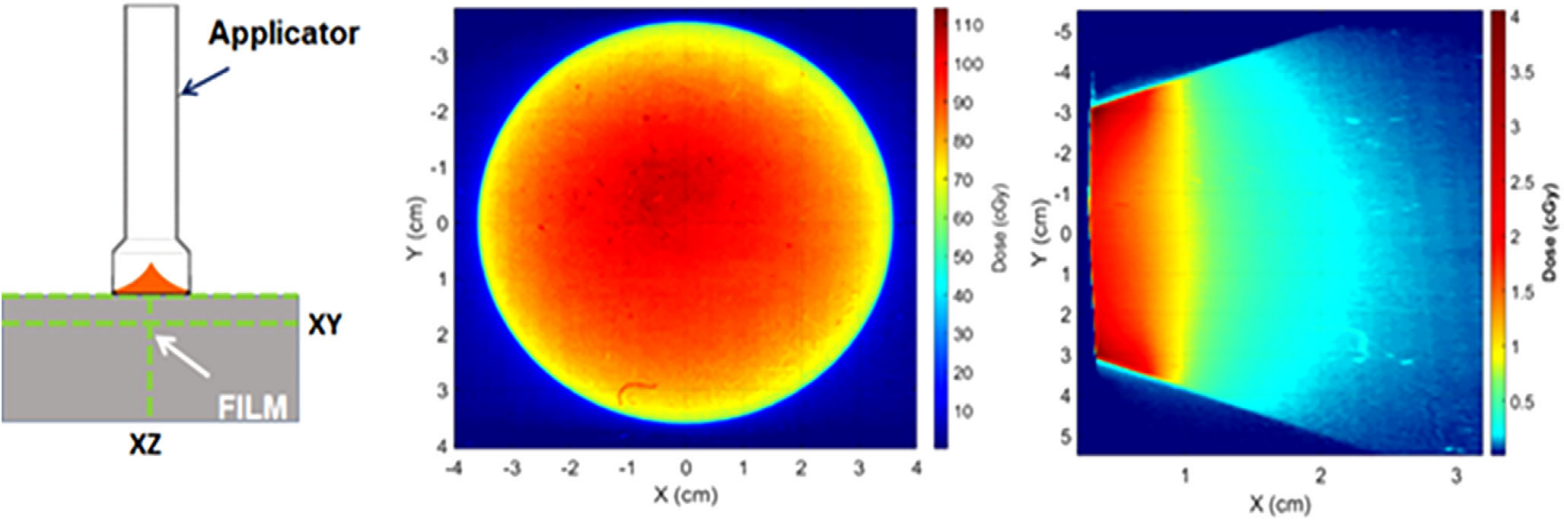
Solid water measurement set‐up with radiochromic films, along with measured 2D distribution for a 6‐cm diameter flat applicator in the *XY* and *XZ* planes.

The characterization of the dose from the flat and surface applicators requires measurements of the following beam properties: (1) depth‐dose variation, (2) surface dose, (3) dose distribution, and (4) beam penumbra. Several dose characterization measurements of the flat and surface applicators have been reported in the past with relatively good agreement between authors.^29^, ^37^, ^38^ The 1‐dimensional dose distribution can be measured with a thin‐window parallel plate ion chamber along the central axis of the applicator in the Zeiss water phantom. The surface dose and 2D dose distributions along the central‐axis and perpendicular to the beam path can be estimated in water^38^ or in solid water.^37^ Figure 4 shows an example measurement set‐up for solid water acquired data with films and example results for a 6 cm FLAT applicator for surface dose and 2D dose variation with depth.

#### 6.3.3 Validation of TPS

**FIGURE 5:**
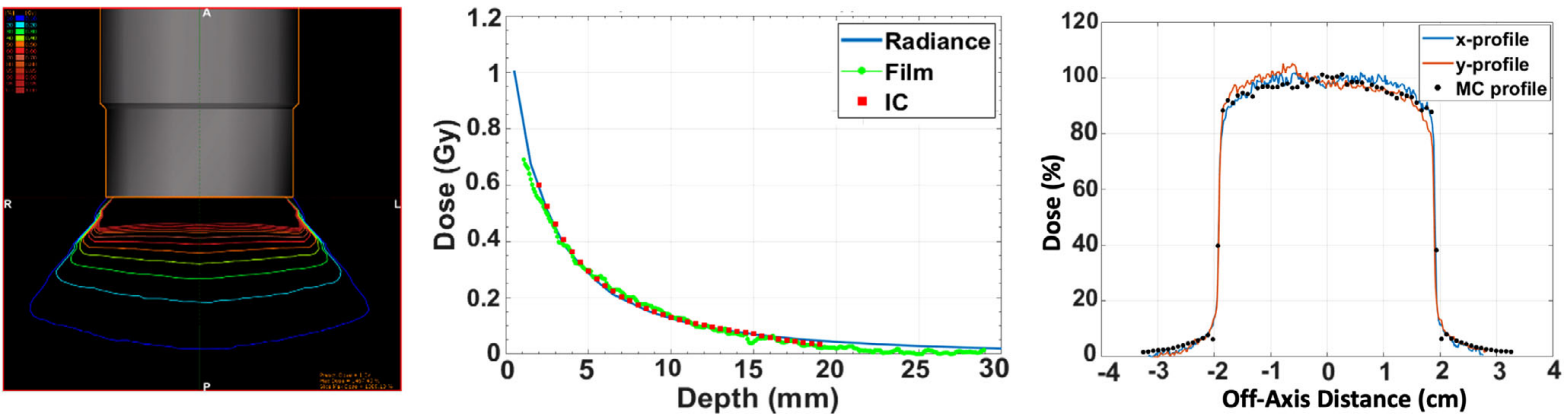
(a) Radiance treatment planning system (TPS) calculated dose distribution for a 4‐cm diameter flat applicator. (b) central axis depth‐dose comparison between ion chamber, film, and TPS calculation for a 4‐cm diameter surface applicator. (c) 1D profiles at 10‐mm depth for a 3‐cm diameter flat applicator illustrating the degree of agreement between Radiance TPS calculation and film measurements.

The source and applicator models from the Radiance TPS should be validated against simulated experimental conditions in water or solid‐water phantoms. Figure 5 shows (A) dose distributions from Radiance for a 4‐cm SURFACE applicator, (B) comparison of 1‐dimensional depth‐dose of a 4 cm SURFACE applicator, and (C) 1‐d profile at 10 mm depth for a 3 cm FLAT applicator.

**FIGURE 6:**
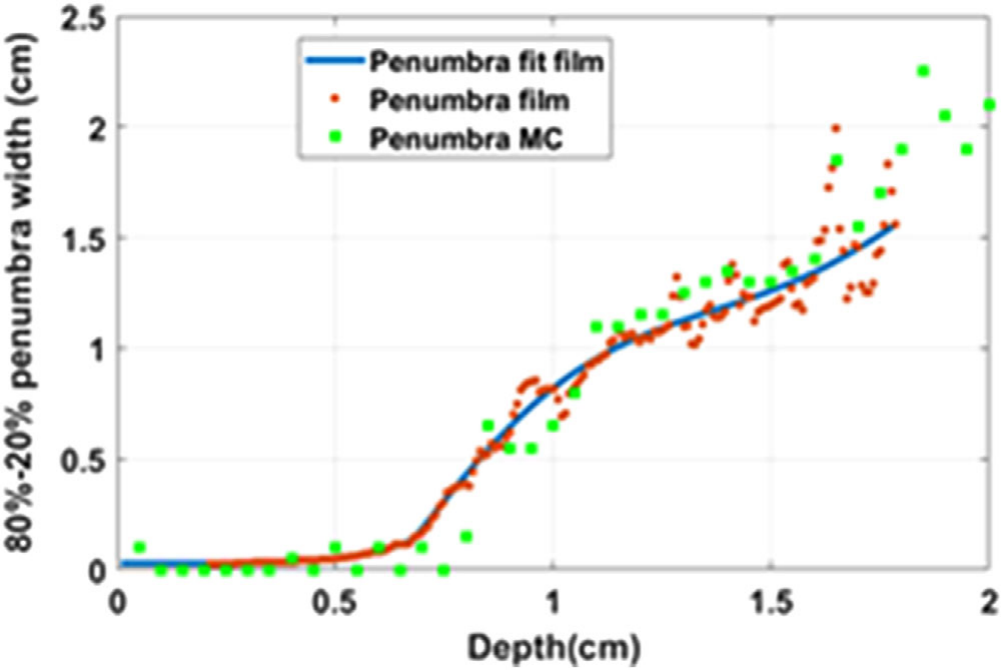
Radiance treatment planning system (TPS) calculated versus measured penumbra (80%−20%) for a 5‐cm diameter flat applicator.

The surface dose varies with applicator type and diameter. Surface applicators are expected to provide homogeneous surface dose. However, the design of the flat applicators can lead to high surface doses at the edge of the applicator. Measured and calculated homogeneity *H* (*D*_max_ */D*_min_) of flat applicators agree within 3% while the depth with maximum homogeneity (*H*_m_) should agree within 1 mm. Expected depth of *H*_m_ ranges between 5 and 10 mm, with *H*_m_ values ranging between 1.02 and 1.13. The depth dependence of the 80%–20% penumbra can be characterized from film measurements. For Surface applicators, the penumbra width sharply increases from the surface then plateaus after reaching 10 mm depth. Flat applicators provide a narrower penumbra (<1mm) up to the depth of *H*_m_, then sharply increase to plateau value at depths of 15 ∼ 20 mm, depending on the applicator size. Figure 6 shows penumbra versus depth plot for a 5 cm FLAT applicator, calculated with Radiance TPS and measured in solid water.

## 7 TG100‐BASED RISK MANAGEMENT ANALYSIS

The utilization of flowcharts and fault‐tree analysis (FTA) based on the AAPM Task Group report 100 (AAPM TG100) can help users better understand and manage the IORT procedure.^43^ As per the AAPM TG 100 report, the authorized medical physicist is responsible for establishing quality assurance standards and workflow for the IORT procedure. The established workflow should be well understood by all members of the multidisciplinary team of surgeons, radiation oncologists, anesthesiologists, medical physicists, and nurses.

**FIGURE 7:**
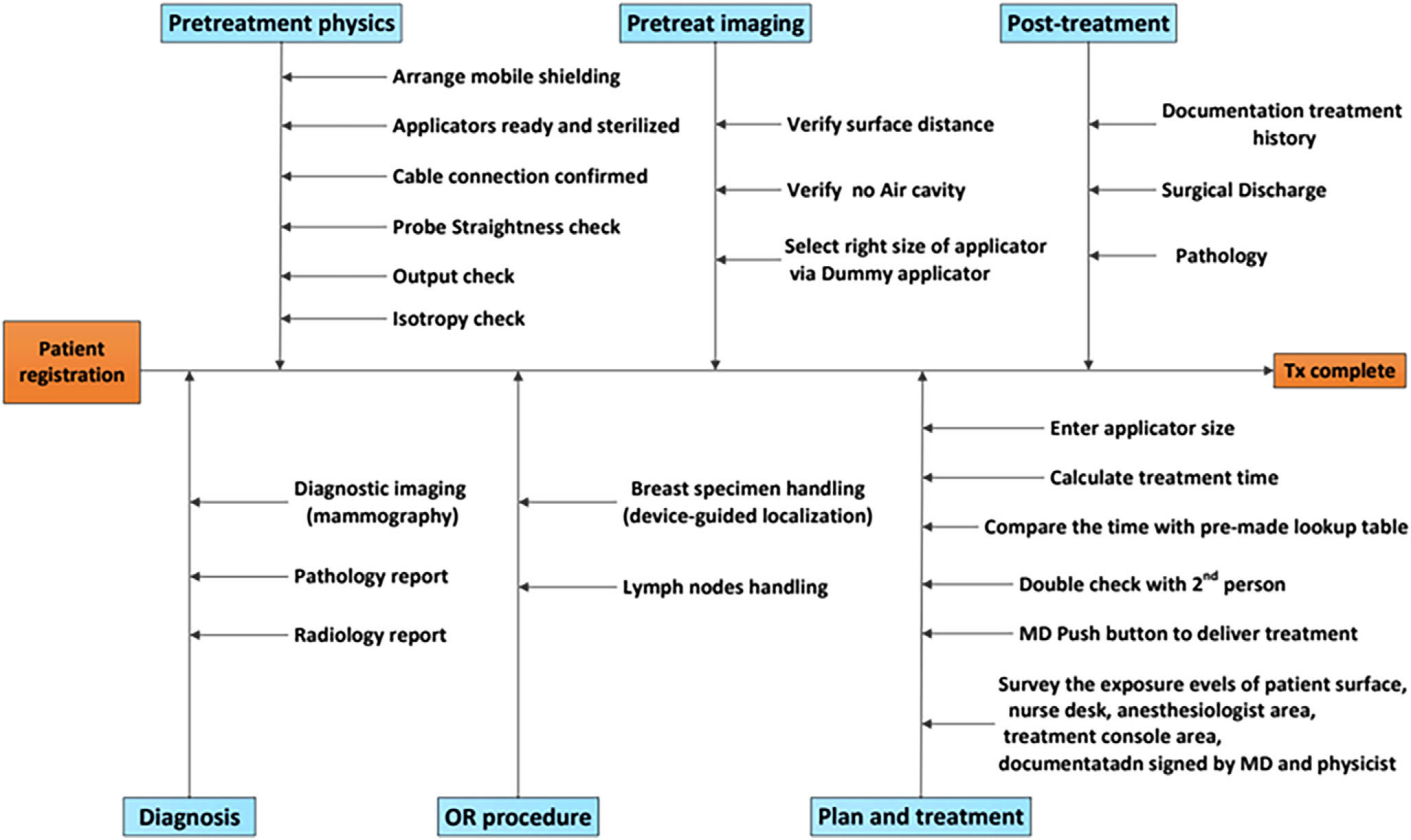
A process map for clinical treatment workflow for TARGIT‐IORT with breast spherical applicator following AAPM TG‐100 guidelines.^43^

An example workflow for INTRABEAM IORT is presented in Figure 7. It should be noted that the workflow details would vary across institutions depending upon available resources, treatment sites, and clinical workload. After personnel training, FMEA^43^ should be performed by the team to identify which processes contribute the greatest risk among the possible failure modes.

**FIGURE 8:**
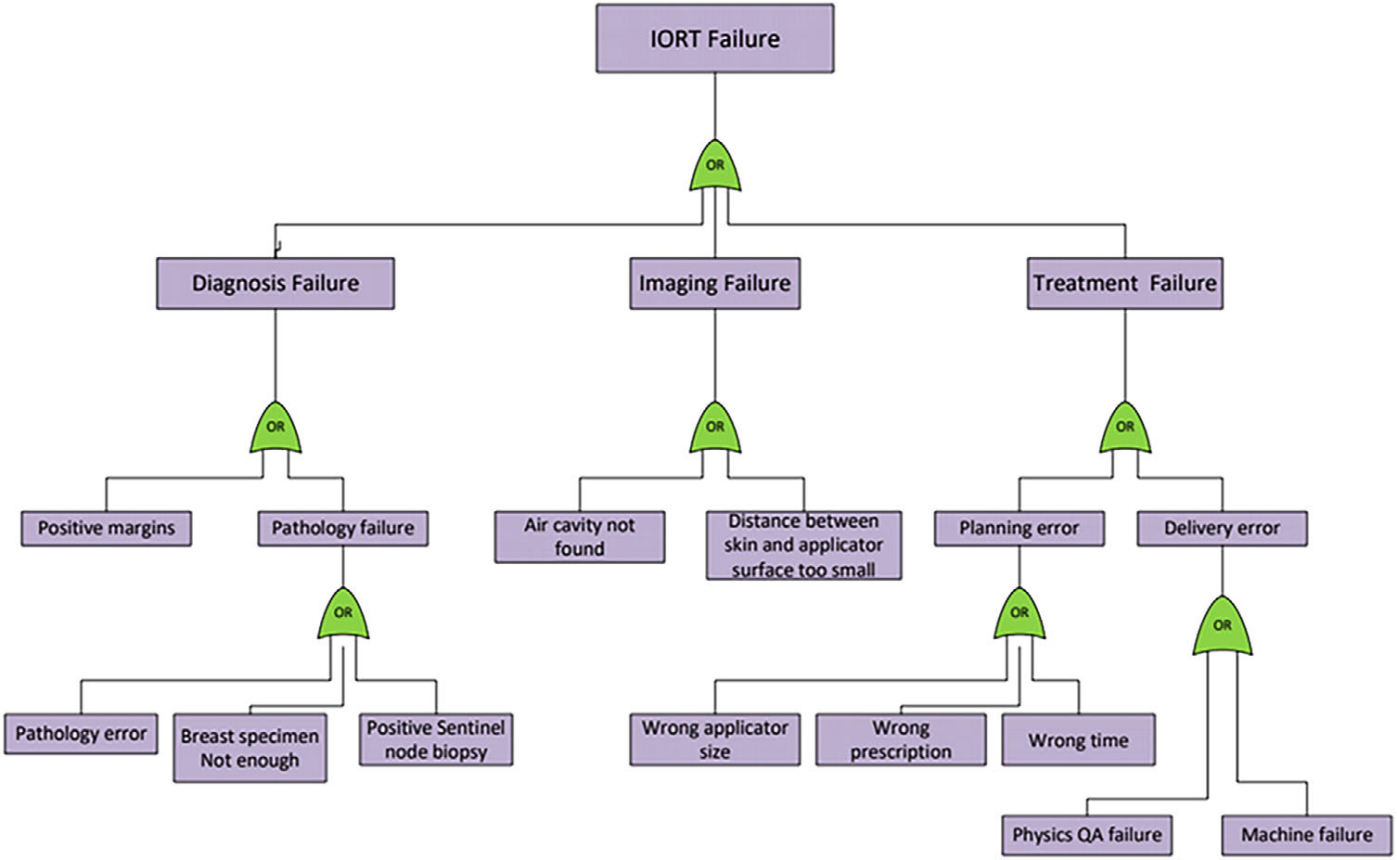
TARGIT IORT fault tree analysis (FTA) following AAPM TG‐100 guidelines.^43^

Figure 8 shows an FTA prepared for an INTRABEAM IORT case. The fault tree begins with the potential failure modes that could result in a failed/abandoned treatment due to either imaging or treatment failure. As illustrated in Figure 8, the sources for each potential failure mode were investigated and traced back to the underlying causes. Actions to address the potential failure modes can be either to eliminate the causes that can start error propagation along the branch of the fault tree or to interrupt the failure progression by setting an intervention along the branch. Both strategies can be effective.

However, specific errors outlined in this example may not occur at other institutions, but there could be other errors that may lead to a treatment failure. It should be noted that most radiotherapy risk analyses are prospective models of planning and delivery that are based on the collective experience, expert knowledge, and expectations of the treatment team members engaged in the analyses.

## 8 IORT CURRENT STATUS AND FUTURE DIRECTIONS

Currently, there are two main modes of IORT delivery and these depend on the type and energy of radiation used: (a) electron IORT (IOERT) and (b) x‐rays IORT (kV IORT). IOERT uses megavoltage electrons with energies between 4 and 12 MeV generated from a mobile electron accelerator, that is, equipped with beam‐shaping beveled applicators to conform radiation to the target shape (IntraOp Mobetron, IntraOp Medical Corporation, Sunnyvale, CA).^3^, ^62^ Beveled applicators allow larger target coverage but at the expense of a less uniform dose and reduced beam penetration in tissue. The electron energy is selected to cover the intended target with a 90% isodose plus a 0.1–0.5 cm safety margin. Typically, bolus is used to increase the surface dose and the largest available applicator (≥2 cm larger than target size) that can be accommodated in the treatment region is employed to avoid lateral geometric miss of the target.

kV IORT may be delivered either with a robotic XRS equipped with applicators (INTRABEAM) as described above or with a miniaturized XRS inside a balloon catheter (Xoft Axxent). The Axxent System (Xoft Inc., Fremont, CA), consists of a miniaturized electronic XRS that is placed inside a flexible probe, a balloon applicator, and a controller to guide the source. The source is a disposable small x‐ray tube that measures approximately 2.2 mm (diameter) × 15 mm (length) and has an operating potential of 50 kV. The controller allows stepping of the XRS to preprogrammed dwell positions in the applicator. Compared to INTRABEAM, the main differences of the Xoft device are that in the latter the source operates within a flexible catheter similar to high dose rate (HDR) brachytherapy, has a higher dose rate, a slower dose fall‐off in the surrounding tissue, and has limited lifetime. A detailed description of the Xoft device is outside the scope of this work and the interested reader is referred to published literature.^2^, ^3^, ^63^

In the following sections, we present some of the clinical challenges and potential research opportunities as they relate to the INTRABEAM IORT system.

### 8.1 CT‐based treatment planning

The most recent INTRABEAM 600 system is equipped with a Radiance treatment planning system (TPS).^59^ Radiance TPS provides a comprehensive, well‐integrated set of treatment planning tools, including image visualization, contouring, Monte Carlo and pencil beam dose computation, DVH calculation, and reporting. Computerized treatment planning also provides accurate description of target and normal tissue dose distribution. This is particularly important as the IORT use is expanding in nonstandard treatment sites or geometries. The dose may be significantly altered due to applicator misalignment and the presence of tissue inhomogeneities, such as, air‐gaps or metal implants. Furthermore, a 3D treatment planning system can enable standardization of IORT practice across different institutions leading to more uniform prescription dose and evaluation of treatment outcomes. Like other Monte Carlo and hybrid Monte Carlo‐based TPS, the computational time is a noteworthy limitation.^59^, ^64^

Simple dose‐rate tables have served well to relate the source output with the prescribed dose and the required irradiation time. This was used successfully within the realm of breast IORT and TARGIT studies where this generally simple calculation can lead to reproducible, well‐standardized irradiation doses. Moreover, it has the benefit of a fast, self‐contained IORT system, that is, portable and amenable to fast treatment planning in the operating room where the treatment planning team has limited time to plan treatment. In situations where tissue composition can be ignored and the applicators are in standard geometries, there is usually no need for 3D treatment planning. Clinical studies have shown that the dose fall‐off is generally such that the organs at risk can be protected by simple mechanical manipulation of tissues, such as adding wet gauze to create separation of critical tissues from the applicator surface. The clinical results confirm that this method works and the side‐effects are acceptable.

Development and widespread utilization of model‐based dose calculation algorithms for electronic brachytherapy (eBT) and INTRABEAM in particular are necessary steps in an effort to compare dose‐outcome relationships among different institutions and radiotherapy options. The variability in tissue composition plays a role in the relative biological effectiveness of the beam quality used. Variation in RBE of up to 1.6 can be seen depending on the tissue type, and this value will change depending on the size of the applicator. Based on radiobiological modeling with the use of equivalent uniform dose and modified linear quadratic model, Schwid et al. showed that a uniform 20 Gy prescription dose for breast IORT may need to be adjusted depending on individual patient's cancer cell infiltration distance.^65^

To accurately estimate the combined dose received by the tumor bed and the organs at risk in adjuvant IORT+EBRT treatment protocols, advanced dose calculations based on 3D volumetric imaging are necessary.

It is expected that with the availability of in‐room imaging systems, there would be greater role of image‐guided radiation therapy for IORT cases. There are several imaging systems available, for example, BrainLab Mobius imaging (Airo 32), Neurologica CT on wheels, Ziehm Imaging, Medtronic CT, and Paracelsus.^66^ Each of these systems is capable of providing in‐room CT imaging. Next, images must be imported into the TPS for planning purposes. To make the process efficient, a treatment plan may be created on simulation imaging (pre‐OR) and then deformed to in‐room CT images. Subsequently, any changes to the plan may be made as needed.

Another important role of in‐room planning can be to aid in independent check of delivered dose as well as confirmation of applicator position in conformance with the treated area. The TPS can also account for heterogeneity corrections caused by the presence of air pockets or bone/high Z materials.^67^ A recent study reports on the use of image guidance in IOERT for a rectal patient. The authors successfully acquired CBCT images with a large bore (102 cm) mobile CT scanner (ImagingRing, medPhoton GmbH). These images were next used with Radiance TPS for real‐time dose calculation. The authors claim that the use of in‐room imaging allowed them to make needed corrections to applicator placement and revise dose prescription to achieve desired target dose.^68^

In‐room CT imaging presents several technical and clinical challenges in the OR. A promising approach that bypasses these difficulties is the use of photogrammetry: acquiring 2D camera images of the surgical site to reconstruct 3D anatomy. These images when coupled with a treatment planning system can yield real‐time dose distribution in the OR. In a recent study by Lozares‐Cordero et al., the authors validated and successfully applied their model to 15 patients with soft‐tissue sarcoma.^69^

### 8.2 Prescription dose implications

Treatment planning with INTRABEAM has been mainly performed via lookup tables. Two dose‐rate tables provided by Zeiss, are x‐ray tube specific and are based on physical measurements performed in a water phantom as a function of distance from the source, *r* (mm). The two dose‐rates, “Calibration V4.0″” and “TARGIT”, stem from the use of two different calibration formalisms. Whereas the “Calibration V4.0″” formalism is more accurate,^70^ the use of the “TARGIT” formalism (based on older calibration protocol) persists in most clinics that treat breast patients. Zeiss attributes these calibration differences to the use of different ion chambers (PTW 23342 vs. PTW34013) their holders and calibration protocols (exposure vs. air‐ Kerma).^3^, ^70^

With the current availability of two FDA‐approved electronic brachytherapy sources on the market, it is becoming increasingly evident that accurate dose reporting is needed. Credentialing of clinical brachytherapy trials relies on dose consistency, accurate dose formalism, and traceability. Watson et al. used Monte‐Carlo‐based calculations to validate the dose in water from the INTRABEAM and Xoft Axxent systems.^71^, ^72^ They reported that, for a nominal TARGIT prescription dose of 20 Gy, the Monte Carlo calculated dose to water at the INTRABEAM applicator surface ranges from 25.2 to 31.7 Gy, depending on the size of the applicator used. The work of Watson et al. demonstrates that despite a nominal prescription of 20 Gy, the delivered dose to the patient will depend on the IORT system used and the size of the chosen applicator. The effect is stronger at smaller distances from the source (30% at the surface of a 1.5 cm spherical applicator) and becomes weaker at large distances (14% at the surface of a 5 cm spherical applicator).^70^, ^71^ It would seem desirable to continue to use the dose prescriptions based on the TARGIT protocol in light of growing clinical evidence and rationale for the use of INTRABEAM in breast IORT. However, a common dosimetry protocol and NIST traceable primary standard would ensure consistency in absorbed dose calculation and inter‐comparison of results with other commercial IORT systems.

Another important point to consider is that while a single TG‐43‐based dose formalism would standardize the electronic brachytherapy (eBT) dosimetry, the homogenous medium such as water does not mimic the properties of human tissues well in the low‐energy range of x‐rays typical of eBT sources.^42^, ^67^ Other sources of dose uncertainty may be related to blood and fluid buildup over target, air gaps, and nonconformance of applicator to target tissue. At these low kV energies, the mass attenuation and mass energy absorption coefficients exhibit large differences due to sizable photoelectric cross section, which is approximately proportional to the cube of the atomic number (*Z^3^*) and inversely proportional to the cube of the photon energy cubed (*E^3^*). Hence, accurate knowledge of tissue type (electron density) and the atomic number distribution of the material is crucial. The AAPM Task Group report 182 recommends that the radiation transport be performed in the heterogeneous medium and that the dose to the local medium be reported along with the TG‐43 calculated doses because the dose error can be up to a factor of 10 if heterogeneity and scatter conditions are ignored.^42^ TG‐182 discusses the importance of assigning tissue composition and mitigating the effect of CT image artifacts in eBT dose calculation techniques.

## 9 SUMMARY OF RECOMMENDATIONS

Medical physicists are an important part of a multidisciplinary team needed to perform IORT in the clinic. They should pay due diligence to the following specific tasks when implementing an IORT brachytherapy program:

1. Commission and implement the IORT system including patient treatment, and post‐treatment verification and documentation to ensure correct source and system functionality (Section 3).
2. Examine all equipment involved in IORT for QA and for patient treatment to create appropriate documentation and checklists (Section 4).
3. Prepare a quality management program that includes all stakeholders in the clinic. In addition to building processes, this includes creating a workflow diagram and evaluating potential failure modes for each step from machine QA to post‐treatment evaluation (Sections 4 and 7). The example workflow presented in Figure 7 can aid in developing workflow evaluation.
4. Be trained on the specific treatment workflow to deliver high‐quality patient care (Section 5).
5. Estimate staff and public exposures before implementation of an IORT brachytherapy program for a specific anatomic site (Sections 3 and 5).
6. Establish radiological safety procedures and provide training to surgical staff (Sections 4 and 5).
7. Utilize a written directive that includes IORT prescription dose, treatment depth, and applicator size (Section 5).
8. Develop a method of secondary check of treatment times for available IORT applicators and intended treatment sites. These treatment times may be obtained from a lookup table for anatomical site, prescription dose, and treatment depth (Section 5).

## AUTHOR CONTRIBUTIONS

All authors contributed in the design, data collection, manuscript preparation, and its final review and editing.

## CONFLICTS OF INTEREST STATEMENT

Portions of this work were presented at the 2022 Annual TARGIT Collaborative Group (TCG) Meeting in Orlando Florida.

## Figures and Tables

**FIGURE A1‐1:**
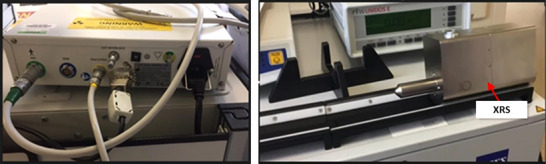
(a) Connection of x‐ray source and photo diode array (PDA) power cables. (b) X‐block and x‐ray source on rail alignment guide.

**FIGURE A2‐1:**
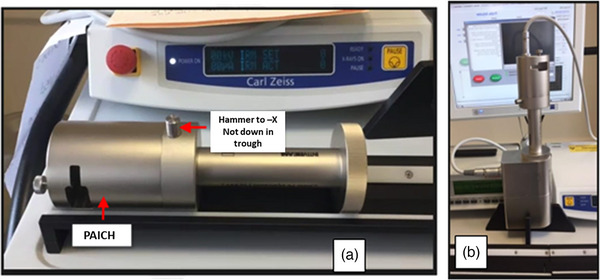
(a) Probe adjuster and ion chamber holder (PAICH) module. (b) x‐ray source (XRS) and PAICH in position for probe adjuster test.

**FIGURE A2‐2:**
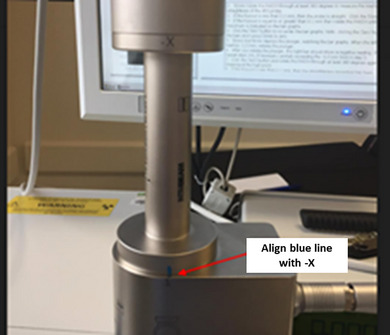
Alignment of photo diode array (PDA) module for Dynamic Offset adjustment.

## APPENDIX 1

### Pre‐treatment QA

#### System preparation

Place the Zeiss Intrabeam cart in a room or area with minimum distractions to perform QA. Unwrap the cable from the back of the unit and plug the Zeiss computer into an uninterruptable power source (typically a red outlet in the OR). To start the unit, first power on the electrometer, second, the PRS control unit, and then the computer monitor. Plug in the color coded XRS and PAICH/PDA cables into the back of the electrometer (Figure A1‐1a). Set up x‐ray source (XRS) flat onto the rail guide and assemble the X block stand on top of the IORT cart. Depending on the cart design, ensure that the trough/sled is on a level surface and is not tilted (Figure A1‐1b).

Next, log into the system and choose “Start Communication” on the screen. Select the “System Quality Assurance” tab to start the QA procedures, then “select XRS”, and the appropriate XRS serial number from the drop down menu. Next, it is recommended to confirm the date and time on the treatment unit. The unit operates with a backup battery, and users have reported a slow drift of the system time. If the system does not display appropriate date/time, the user is encouraged to contact Zeiss to reset the system clock. Battery drifts have been reported to cause the system to not recognize that the QA was completed within the 36‐h window at the time of treatment, leading to procedure delays. The physicist should also inspect the QA cables for the XRS and the PAICH/PDA. Users have reported exposure/fraying of the cable optics due to improper handling (torqueing or pulling on cable).

## APPENDIX 2

### Daily QA

#### Probe adjuster test—Recommended

The probe adjuster test uses an LED and photodiode to correlate the signal from the light reflected by the source probe for centering of the probe. A small “hammer” is located at the side of the PAICH module that can be used to apply a force to straighten the probe. The steps to follow for these tests are described below:

1. Slide the XRS into the PAICH using the trough and connect cables appropriately with the hammer. facing away from the trough to prevent probe deflection (Figure A2‐1a).
2. Place the XRS and PAICH into X block stand (Figure A2‐1b).
3. Click on the blue zero button on the computer screen to zero the source position.
4. While holding the PAICH cable about 1 ft above the PAICH, rotate the PAICH 360° to check the tip run out. Values may fluctuate on the screen based on cable position. Source runout needs to be <0.1 mm.

#### Dynamic Offsets—Recommended

The Dynamic Offset adjustment automatically steers electrons down the drift tube to minimize source emission anisotropy. It should be performed after probe adjustment to ensure the centering of the source emission. The PDA module is used to control the source isotropy in orthogonal directions. It is important to align the PDA module along the X and Y directions indicated on the source (Figure A2‐2). The Dynamic Offset steps are described below:

1. Remove PAICH from XRS using the trough.
2. Slide XRS into PDA using the trough and connect cables appropriately.
3. Place the XRS and PDA into X block stand.
4. Press Dynamic Offsets button on the computer screen.
5. “Filament not ready” will be displayed as the system prepares for beam on.
6. Align blue line with –X (see Figure A2‐2).
7. Press Start on screen once ready. Radiation is on.
8. When the test is finished, the message window will display “successfully completed”.

#### PDA Source Check (or Isotropy check)—Mandatory

The PDA source check is mandatory, as it measures variation of the source output with the internal radiation monitor (IRM), as well as ensuring that the source isotropy is within user and manufacturer tolerance prior to initiating a patient treatment. The source emission is verified along the orthogonal X/Y directions with four photodiodes against a single photodiode placed in front of the probe tip on the *Z* axis. It is also important to align the PDA module along the *X* and *Y* directions indicated on the source (Figure A2‐2) for this test. The steps to run the PDA test are described below:

1. Press PDA Source Check on computer monitor.
2. Press Start.
3. IRM deviation is within 10%−15%.
4. Update history = YES.

#### PAICH Output Check—Mandatory

The PAICH output check measures the relative source output in air with a parallel plate ion chamber (PTW TN 23342) inserted at the top of the PAICH module (Figure A2‐1b). The measured relative output difference is used by the system control unit to adjust treatment times accordingly. The steps to run the PAICH output check are listed below:

1. Remove PDA from XRS using the trough.
2. Slide XRS into PAICH using the trough and connect cables appropriately.
3. Place the XRS and PAICH into X block stand.
4. Insert the parallel plate ion chamber into PAICH.
5. Pull up on pin to secure the ion chamber in PAICH.
6. Press PAICH Output Check.
7. Push Start.
8. Output should be within 5%−10%.
